# Maternal Residential Proximity to Major Roadways and Pediatric Embryonal Tumors in Offspring

**DOI:** 10.3390/ijerph15030505

**Published:** 2018-03-13

**Authors:** Shwetha V. Kumar, Philip J. Lupo, Lisa A. Pompeii, Heather E. Danysh

**Affiliations:** 1 Department of Epidemiology, Human Genetics and Environmental Sciences, University of Texas Health Science Center at Houston, 1200 Pressler Street, Houston, TX 77030, USA; shwetha.v.kumar@uth.tmc.edu (S.V.K.); lisa.pompeii@uth.tmc.edu (L.A.P.); 2 Department of Pediatrics, Section of Hematology-Oncology, Baylor College of Medicine, One Baylor Plaza, Houston, TX 77030, USA; Philip.Lupo@bcm.edu

**Keywords:** traffic-related air pollution, childhood cancer, neuroblastoma, Wilms tumor, retinoblastoma, hepatoblastoma

## Abstract

The environmental determinants of pediatric embryonal tumors remain unclear. Because of the growing concern over the impact of exposures to traffic-related air pollution on pediatric cancer, we conducted a population-based study evaluating the impact of maternal residential proximity to major roadways on the risk of pediatric embryonal tumors in offspring. We identified children diagnosed with neuroblastoma, Wilms tumor, retinoblastoma, or hepatoblastoma at <5 years of age from the Texas Cancer Registry and selected unaffected controls from birth certificates. Two residential proximity measures were used: (1) distance to the nearest major roadway, and (2) within 500 m of a major roadway. Logistic regression was used to estimate the adjusted odds ratio (aOR) and 95% confidence interval (CI) for each proximity measure on pediatric embryonal tumors. The odds of an embryonal tumor were increased in children born to mothers living within 500 m of a major roadway (aOR = 1.24, 95% CI: 1.00, 1.54). This was consistent for most tumor subtypes, with the strongest associations observed for unilateral retinoblastoma (aOR = 2.57, 95% CI: 1.28, 5.15, for every kilometer closer the mother lived to the nearest major roadway). These findings contribute to the growing evidence that traffic-related air pollution may increase risk for certain pediatric tumors.

## 1. Introduction

Embryonal tumors are a group of rare heterogeneous malignancies originating from undifferentiated cells in embryonic tissue, and develop primarily in children and adolescents. Outside of the central nervous system (CNS), the major embryonal tumor subtypes include: (1) neuroblastoma, (2) Wilms tumor, (3) retinoblastoma, and (4) hepatoblastoma. While advances in therapy have led to improvements in five-year survival for children diagnosed with an embryonal tumor (>70% for all non-CNS embryonal tumor subtypes [1,2]), survivors often develop serious adverse health conditions as a result of their treatment, including second malignant neoplasms, cardiovascular disease, hearing loss, and endocrinopathies [2,3]. Germline mutations in known cancer predisposition genes (e.g., *WT1* gene [chromosome 11p13] or *WT2* gene [chromosome 11p15] in Wilms tumor, and *RB1* gene [chromosome 13q14] in bilateral retinoblastoma) are responsible for approximately 10% of pediatric embryonal tumors, however, most cases are sporadic with no known etiology [4,5,6,7]. As the incidence of embryonal tumors peaks in the first three years of life, it is suspected that exposures in utero and in early life may be important for the development of these tumors [2,8].

There is an increasing concern over the potential role that early-life exposures to traffic-related air pollution may have on the development of pediatric embryonal tumors. Automobile emissions are composed of many compounds that are known to be carcinogenic to humans, including polycyclic aromatic hydrocarbons (PAH), benzene, formaldehyde, 1,3-butadiene, and particulate matter (PM_2.5_ and PM_10_) [9,10]. The highest ambient concentrations of these harmful compounds from automobiles are within 500 m of major roadways, an area where an increasing proportion of the population resides [9,11]. There is recent evidence suggesting that living near a major roadway may increase cancer risk in children, specifically for leukemia and CNS tumors, including CNS embryonal tumors [12,13]. However, little is known about the role of traffic-related air pollution and living near major roadways on risk for non-CNS pediatric embryonal tumors.

To address this gap, we conducted a population-based case-control study to evaluate the association between maternal residential proximity to major roadways and non-CNS pediatric embryonal tumors in offspring. Specifically, we assessed associations with neuroblastoma, retinoblastoma, Wilms tumor, and hepatoblastoma using data from the Texas Cancer Registry (TCR), one of the largest cancer registries in the United States (U.S.) that captures cancer cases from both large rural and metropolitan areas with high and variable levels of traffic-related air pollution.

## 2. Materials and Methods

### 2.1. Study Population

We identified children born in Texas between 1 January 2003 and 31 December 2009 who were diagnosed with an embryonal tumor at <5 years of age during the same period, as the highest incidence of pediatric embryonal tumors is in this age group. Cases were ascertained from the TCR, which is a statewide population-based cancer registry administered by the Texas Department of State Health Services (TX DSHS) and was Gold certified by the North American Association of Central Cancer Registries during the study period. We used the *International Classification of Childhood Cancer, Third Edition* (ICCC-3), and the *International Classification of Diseases for Oncology, Third Edition* (ICD-O-3), to define the embryonal tumor subtypes: neuroblastoma (ICD-O-3 histology code 9500), retinoblastoma (ICCC-3 group V), Wilms tumor (ICCC-3 group VIa.1), and hepatoblastoma (ICCC-3 group VIIa) [14]. Retinoblastoma cases were classified as either (1) unilateral, if only one eye was involved, or (2) bilateral, if both eyes were involved. Only cases diagnosed with an embryonal tumor as their first primary malignancy were included. For each case, the TCR also provided information on age at the time of diagnosis as well as used probabilistic linkage to link the cancer registry record to Texas birth certificates [15].

Unaffected control subjects were randomly selected among the birth certificate records of those born in Texas during the same period as cases (2003–2009) and were not included in the TCR. Controls were frequency matched to cases on birth year at a ratio of five control subjects for every embryonal tumor case. For all study subjects, we obtained the geocoded address of the maternal residence at the time of delivery (i.e., at the time of the child’s birth), as well as other infant- and maternal-related information, from the birth certificate records. The Institutional Review Boards of the Baylor College of Medicine, the University of Texas Health Science Center at Houston, and the TX DSHS approved the study protocol (TX DSHS IRB 14-034).

### 2.2. Exposure Assessment

To measure maternal proximity to major roadways, we used the Texas roadway network StratMap for 2006, the midpoint of the study period (2003–2009) [16]. Exposure to traffic-related air pollution was assessed using two surrogate measures, each a proximity measure of the maternal residence to major roadways: (1) continuous distance of the residence to the nearest major roadway, and (2) the presence of a major roadway within a 500-m radius of the residence. As previously demonstrated, the 500-m area adjacent to a major roadway has the highest concentrations of primary air pollutants from traffic emissions, therefore, this was selected as the area of interest to assess exposure [9]. Roadways designated by the U.S. Census Bureau as interstate, state, county, or toll highways (i.e., Feature Class Codes A1, A2, or A3) were defined as a “major roadway” [17]. The longitude and latitude data points for the maternal residential address at the time of delivery were used to define the residential location.

A geographic information systems (GIS) approach was used to assess exposure. Spatial analysis was employed using ArcGIS, version 10.0 (Environmental Systems Research Institute Inc., Redlands, CA, USA). The distance of the maternal residence to the nearest major roadway was measured using the Euclidean distance (i.e., ‘as the crow flies’) and was modeled as a continuous variable (kilometers (km)). Buffer analysis was used to determine if a major roadway was within a 500-m radius from the maternal residence; exposure was modeled as a dichotomous variable based on whether a major roadway fell within the 500-m radius area around the maternal residential location (yes or no).

### 2.3. Covariates

Potential covariates were selected a priori and included infant characteristics (sex, birth weight, gestational age, and season of birth) and maternal characteristics (race/ethnicity, age, education level, and smoking status at the time of delivery) [7,18,19,20,21,22,23,24]. Information on infant and maternal characteristics was obtained from birth certificate records. Information on household income was not available from birth certificates, therefore, we used area-level poverty measured at the census tract level as a proxy measure of socioeconomic status. Census tracts where <15% of households had an annual income below the poverty level were defined as having “low area-level poverty”; census tracts where ≥15% of households had an income below the poverty level were defined as having “high area-level poverty”. Census tract-level estimates on the proportion of households with an income below the poverty level in addition to information on census tract-level urban-rural status was obtained from the 2000 U.S. Census.

### 2.4. Statistical Analysis

Descriptive statistics were calculated for infant and maternal characteristics as well as area-level factors, including poverty and urban status, for both cases and controls. The distribution of the exposure (i.e., means and frequencies) among cases and controls was calculated for each of the residential proximity measures. Logistic regression was used to examine the association between each of the residential roadway proximity measures and each embryonal tumor subtype, as well as all embryonal tumors combined. Unadjusted and adjusted odds ratios (OR and aOR, respectively) and 95% confidence intervals (CI) were calculated to examine associations. Covariates included in the final adjusted models were selected a priori, and included: birth weight, gestational age, maternal age at delivery, maternal race/ethnicity, and area-level poverty. Birth year was also included as a covariate in all regression models as this was the matching factor for cases and controls. Associations were considered statistically significant if the *p*-value was <0.05. All statistical analyses were conducted using STATA, version 15.1 (StataCorp LP, College Station, TX, USA).

## 3. Results

### 3.1. Characteristics of Cases and Controls

Out of the 571 embryonal tumor cases identified, neuroblastoma (*n* = 252 (44.1%)) was the largest group, followed by Wilms tumor (*n* = 143 (25.0%)), retinoblastoma (*n* = 121 (21.2%)), and hepatoblastoma (*n* = 55 (9.6%)). Among those with retinoblastoma, the majority were unilateral cases (*n* = 89 (62.2%)). Most cases (68.8%) were diagnosed with an embryonal tumor before one year of age. Table 1 presents the distribution of infant, maternal, and demographic characteristics among cases within each of the embryonal tumor subtypes, as well as among control subjects. Overall, the majority of embryonal tumor cases were male (55.0%), with the exception of Wilms tumor cases, which were predominantly female (54.6%), and unilateral retinoblastoma cases, which did not vary by sex. Compared to controls, embryonal tumor cases were more likely to be born pre-term (<37 weeks gestation) (18.2% of cases vs. 12.3% of controls) and of low birth weight (<2500 g) (12.4% of cases vs. 8.8% of controls).

With the exception of bilateral retinoblastoma cases, embryonal tumor cases were more likely to be delivered by mothers who were ≥30 years old compared to control subjects (41.5% of case mothers vs. 32.2% of control mothers). Mothers of control subjects were more likely to be Hispanic (47.2%) than any other race/ethnicity. Likewise, the highest proportion of mothers of cases with unilateral retinoblastoma (43.8%), bilateral retinoblastoma (56.3%), and hepatoblastoma (56.4%) were Hispanic, while children with neuroblastoma (52.7%) and Wilms tumor (44.8%) were more likely to be born to non-Hispanic white mothers than any other race/ethnicity. Control mothers (58.9%) and case mothers (63.6%) predominantly lived in areas with low poverty, however, mothers of hepatoblastoma cases were more likely to live in areas with high poverty (54.5%).

### 3.2. Continuous Distance to Nearest Major Roadway

Associations between the continuous distance of the maternal residence to the nearest major roadway and embryonal tumors in offspring are presented in Table 2. These results indicate that for every one km closer a mother lived to a major roadway at the time of delivery, the odds of her offspring having an embryonal tumor increased by 13% (95% CI: 0.97, 1.32). Notably, this association was strongest with cases diagnosed with unilateral retinoblastoma (aOR = 2.57, 95% CI: 1.28, 5.15). In addition, we observed non-statistically significant weak positive associations with neuroblastoma, Wilms tumor, bilateral retinoblastoma, and hepatoblastoma.

### 3.3. Within 500 m of a Major Roadway

**Figure 1:**
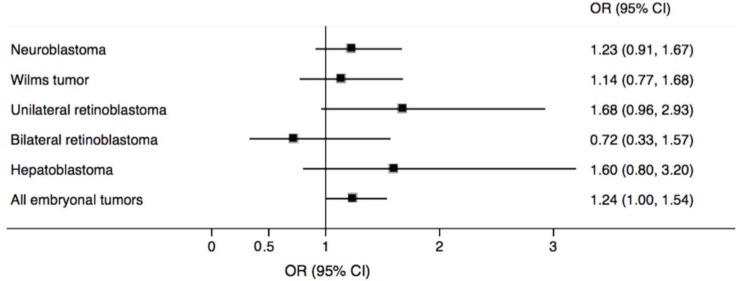
Adjusted odds ratios (OR) and 95% confidence intervals (CI) for maternal residential proximity <500 m from the nearest major roadway and embryonal tumors in offspring. The squares represent the adjusted OR for each tumor type. The horizontal line intersecting each square represents the respective 95% CI.

Associations between maternal residential proximity within 500 m of a major roadway and embryonal tumors in offspring are presented in Table 3 and Figure 1. These results indicate that mothers living <500 m from the nearest major roadway had a 24% greater odds (95% CI: 1.00, 1.54) of having offspring with an embryonal tumor compared to mothers living ≥500 m from the nearest major roadway. Positive associations were observed with unilateral retinoblastoma (aOR = 1.68, 95% CI: 0.96, 2.93), hepatoblastoma (aOR = 1.60, 95% CI: 0.80, 3.20), neuroblastoma (aOR = 1.23, 95% CI: 0.91, 1.67), and Wilms tumor (aOR = 1.14, 95% CI: 0.77, 1.68). However, in each case, the confidence intervals included 1.00. We also observed a non-statistically significant inverse association with bilateral retinoblastoma (aOR = 0.72, 95% CI: 0.33, 1.57).

## 4. Discussion

Overall, we observed that children born to mothers living within 500 m of a major roadway at the time of delivery were more likely to have an embryonal tumor compared to those born to mothers living further than 500 m from the nearest major roadway. This association was strongest for unilateral retinoblastoma where the odds increased 2.6 times for every km closer the mother lived to a major roadway. In addition, we observed positive associations between maternal residential proximity to major roadways and neuroblastoma, Wilms tumor, and hepatoblastoma; however, the small number of cases in each of these tumor subtypes may have limited our power to achieve statistical significance given the estimated effect sizes.

Our findings are consistent with a prior study that assessed associations between air pollution exposures and retinoblastoma using data from the California Cancer Registry. They reported an increased risk in unilateral retinoblastoma (*n* = 65 cases; aOR = 1.44, 95% CI: 0.93, 2.22) for every one interquartile range (IQR) increase in prenatal exposure to PM_2.5_, a carcinogenic air toxic that is highly associated with automobile emissions [25]. Additionally, there is previous evidence suggesting that maternal smoking near the time of conception and during pregnancy may increase the risk of unilateral retinoblastoma in offspring [26,27]. As exposures to smoking and automobile emissions contain many of the same carcinogenic compounds, the mechanisms for carcinogenesis induced from these exposures may be similar. Considering the current evidence, early exogenous exposures, including exposure to air pollution, may be important for the etiology of unilateral retinoblastoma.

Retinoblastoma is the most common intraocular malignancy in children, and typically presents in one of two forms, hereditary or non-hereditary (sporadic). The tumor occurs due to the loss of the tumor suppression functions of the *RB1* gene which is the result of a biallelic mutation [5]. Hereditary retinoblastoma is characterized by at least one inherited mutated copy of the gene from an affected parent with a subsequent “second hit” mutation occurring at post-conception leading to the disease [27]. These cases are predominantly bilateral as the mutation is present in all cells. Bilateral hereditary retinoblastoma can also be due to a de novo germline mutation in *RB1* [27]. In this case, there is no family history of retinoblastoma. Lastly, most children (>70%) with retinoblastoma do not have germline mutations in *RB1* and exhibit the unilateral subtype [27]. In light of the known genetic determinants and etiology of retinoblastoma, it is suspected that exogenous exposures may be important for both the unilateral and bilateral subtypes, however, more so for the unilateral as these children do not have inherited germline mutations in *RB1* [28].

While the specific mechanisms that underlie these associations are unknown, automobile emissions are composed of several hazardous air pollutants, many of which are known human carcinogens [9]. For example, previous animal studies and observational human studies have reported that exposures to PAH, a known carcinogen present in automobile emissions, are associated with the presence of PAH-induced DNA adducts in the blood, including cord blood in newborns, which are known to promote carcinogenesis and increase cancer risk [29,30,31]. In addition, exposures to carcinogenic compounds, including PAH, are known to produce free radicals and induce oxidative stress leading to mutagenesis [32,33]. Given this, it is plausible that exposures to pollutants associated with automobile emissions may promote cancer development in children.

The use of residential proximity to major roadways as a proxy for exposure to traffic-related air pollution may be a limitation of this study. It is typically not feasible to obtain personal exposure levels of pollutants in studies of rare diseases, such as pediatric cancers, and exposures derived from air monitors in Texas are not reliable as these are inconsistently located across the state. However, proximity measures to pollutant sources have been validated as a reliable surrogate for assessing exposures to air pollution [9,34]. Information on traffic volume was not available during the study period. In addition, because of the rare nature of embryonal tumors, the sample sizes in our study were small, particularly for hepatoblastoma (*n* = 55) and bilateral retinoblastoma (*n* = 32), limiting our ability to achieve statistically significant estimates. Our study assessed exposures at the time of birth only, without measuring exposures at other potentially critical developmental periods (e.g., in utero), as Texas birth certificates list only the maternal address at the time of delivery and do not include information on residential history. However, previous studies have suggested that air pollution exposures measured at the time of birth may be an appropriate proxy for exposure measures during the prenatal period, at the time of conception, in infancy, and early childhood [35,36,37]. The study population was limited to include only cases that were diagnosed at <5 years of age, in whom exposures near the time of birth would be most relevant. Lastly, genetic characteristics were not included in this assessment as this information is not available from the TCR.

Despite these limitations, this study has several strengths. We used a population-based sample of pediatric embryonal tumors from TCR, one of the largest cancer registries in U.S. This allowed for the evaluation of associations among rare embryonal tumor subtypes. Furthermore, the study subjects were residents of Texas, which provides a unique backdrop for air pollution studies as this state has some of the highest air pollutant concentrations in the U.S. [38]. Finally, we used residential proximity to a major roadway to assess exposure which may account for the overall exposure to the multiple co-occurring pollutants in traffic-related air pollution. Many air pollution–health effects studies assess exposures to single pollutants; however, individuals are typically exposed to multiple pollutants at one time.

## 5. Conclusions

The results from this study suggest that mothers living within 500 m of a major roadway at the time of delivery may be more likely to have offspring with an embryonal tumor in early childhood compared to mothers living further than 500 m from a major roadway. Moreover, we observed a strong association with unilateral retinoblastoma, which is supported by previous studies [25,26,27]. In addition, while several of the sample sizes in the individual embryonal tumor subtypes were small and most of the effect estimates were not statistically significant, we consistently observed positive associations across all tumor subtypes, apart from bilateral retinoblastoma. These findings underscore the need for further investigation into understanding the potential role that exposures to traffic-related air pollution may have in the development of pediatric embryonal tumors. Future studies must include biomarkers of exposures to air pollutants, as well as an assessment of gene-environment interactions to better understand the role of genetic and environmental factors in the development of pediatric cancers.

## Figures and Tables

**Table 1:** Characteristics of pediatric embryonal tumor cases and control subjects in Texas, 2003–2009.

Characteristic, *n* (%)	All Embryonal Tumors	Neuroblastoma	Wilms Tumor	Retinoblastoma		Hepatoblastoma	Controls
				Unilateral	Bilateral		
Total subjects	571 (100)	252 (44.1)	143 (25.0)	89 (15.6)	32 (5.6)	55 (9.6)	2855 (100)
Infant							
Male sex	314 (55.0)	154 (61.1)	65 (45.4)	46 (51.6)	19 (59.4)	30 (54.5)	1436 (50.3)
Gestational age							
37–42 weeks	424 (74.3)	187 (74.2)	117 (81.8)	64 (71.9)	24 (75.0)	32 (58.2)	2320 (81.3)
<37 weeks	104 (18.2)	45 (17.9)	NS ^3^	14 (15.7)	NS ^3^	NS ^3^	350 (12.3)
>42 weeks	43 (7.5)	20 (7.9)	NS ^3^	11 (12.4)	NS ^3^	NS ^3^	185 (6.5)
Birth weight							
2500–3999 g	464 (81.3)	210 (83.1)	122 (85.0)	73 (82.0)	28 (87.5)	31 (56.4)	2430 (85.1)
<2500 g	71 (12.4)	30 (12.2)	9 (6.4)	10 (11.2)	NS ^3^	18 (32.7)	252 (8.8)
≥4000 g	36 (6.3)	12 (4.7)	12 (8.6)	6 (6.8)	NS ^3^	6 (10.9)	173 (6.1)
Season of birth ^1^							
Summer	124 (22.8)	60 (23.8)	30 (20.9)	17 (19.1)	9 (28.1)	16 (29.1)	732 (25.6)
Fall	144 (26.6)	66 (26.2)	34 (23.8)	29 (32.6)	9 (28.1)	13 (23.6)	721 (25.3)
Winter	143 (26.4)	64 (25.4)	35 (24.5)	25 (28.1)	8 (25.0)	18 (32.7)	673 (23.6)
Spring	131 (24.2)	62 (24.6)	44 (30.8)	18 (22.2)	6 (18.8)	8 (14.6)	729 (25.5)
Age at diagnosis							
≤1 year	393 (68.8)	200 (79.4)	65 (45.5)	61 (68.5)	NS ^3^	36 (65.5)	
>1 year	178 (31.2)	52 (20.6)	78 (54.5)	28 (31.5)	NS ^3^	19 (34.5)	
Maternal ^2^							
Race/ethnicity							
NH White	258 (45.2)	132 (52.4)	66 (44.8)	33 (37.1)	8 (25.0)	21 (38.2)	1031 (36.1)
NH Black	70 (12.3)	26 (10.3)	NS ^3^	NS ^3^	NS ^3^	NS ^3^	353 (12.4)
Hispanic	225 (39.4)	82 (32.5)	54 (38.5)	39 (43.8)	18 (56.3)	31 (56.4)	1348 (47.2)
Other	18 (3.2)	12 (4.7)	NS ^3^	NS ^3^	NS ^3^	NS ^3^	123 (4.3)
Age ^4^							
<20 years	74 (12.9)	34 (13.5)	16 (11.2)	11 (12.4)	8 (25.0)	NS ^3^	414 (14.5)
20 to <25 years	128 (22.4)	54 (21.4)	31 (21.7)	20 (22.5)	6 (18.8)	NS ^3^	793 (27.8)
25 to <30 years	132 (23.1)	55 (21.8)	36 (25.2)	23 (25.8)	8 (25.0)	10 (18.2)	730 (25.6)
30 to <35 years	152 (26.6)	68 (27.0)	36 (25.2)	25 (28.1)	NS ^3^	16 (29.1)	574 (20.1)
≥35 years	85 (14.9)	41 (16.3)	24 (16.7)	10 (11.2)	NS ^3^	7 (12.7)	344 (12.1)
Completed education ^4^							
>HS	293 (51.5)	136 (54.0)	83 (58.0)	40 (45.5)	11 (41.9)	21 (38.2)	1231 (43.3)
Completed HS	137 (24.1)	58 (23.0)	32 (22.4)	23 (26.1)	7 (22.6)	17 (30.9)	790 (27.8)
<HS	139 (24.4)	58 (23.0)	28 (19.6)	25 (28.4)	11 (35.5)	17 (30.9)	822 (28.9)
Neighborhood							
Area-level poverty							
Low poverty	363 (63.6)	175 (69.4)	96 (67.1)	49 (55.1)	18 (56.3)	25 (45.5)	1680 (58.9)
High poverty	208 (36.4)	77 (30.6)	47 (32.9)	40 (44.9)	14 (43.7)	30 (54.5)	1174 (41.1)
Urban status							
Rural	54 (9.5)	22 (8.8)	17 (11.9)	NS ^3^	NS ^3^	NS ^3^	288 (10.1)
Urban	517 (90.5)	228 (91.2)	126 (88.1)	NS ^3^	NS ^3^	NS ^3^	2566 (89.9)

Abbreviations: g, grams; HS, high school; NH, non-Hispanic; NS, not shown; ^1^ Summer, June through August; Fall, September through November; Winter, December through February; Spring, March through May; ^2^ Estimates for maternal smoking are not shown due to small cell sizes. Overall, 7.0% of case mothers and 7.9% of control mothers were smokers; ^3^ Estimates not shown due to small cell sizes (i.e., one cell is *n* ≤ 5) and protection of confidentiality; ^4^ at delivery.

**Table 2:** Associations between continuous distance (km) of maternal residence to the nearest major roadway and embryonal tumors in offspring.

Tumor Type	Mean (SD)			Odds Ratio (95% CI)			
	Cases	Controls		Unadjusted	*p*-Value	Adjusted ^1^	*p*-Value
All embryonal tumors	0.43 (0.59)	0.46 (0.66)		1.06 (0.92, 1.22)	0.443	1.13 (0.97, 1.32)	0.121
Neuroblastoma	0.47 (0.59)	0.46 (0.66)		0.96 (0.80, 1.16)	0.672	1.06 (0.86, 1.30)	0.591
Wilms tumor	0.45 (0.64)	0.46 (0.66)		1.00 (0.77, 1.30)	0.988	1.07 (0.81, 1.43)	0.633
Retinoblastoma							
Unilateral	0.28 (0.31)	0.46 (0.66)		2.52 (1.28, 4.97)	0.008	2.57 (1.28, 5.15)	0.008
Bilateral	0.41 (0.33)	0.46 (0.66)		1.10 (0.60, 2.03)	0.750	1.04 (0.57, 1.90)	0.909
Hepatoblastoma	0.46 (0.80)	0.46 (0.66)		1.00 (0.68, 1.49)	0.982	1.06 (0.72, 1.56)	0.785

Abbreviations: CI, confidence interval; km, kilometer; SD, standard deviation; ^1^ Adjusted for birth year, birth weight, gestational age, maternal race/ethnicity, maternal age at delivery, and area-level poverty.

**Table 3:** Associations between maternal residential proximity within 500 m of a major roadway and embryonal tumors in offspring.

Proximity to Major Roadway	*n* (%)			Odds Ratio (95% CI)			
	Cases	Controls		Unadjusted	*p*-Value	Adjusted ^1^	*p*-Value
All embryonal tumors							
>500 m (ref.)	142 (24.9)	772 (27.0)		1.00		1.00	
≤500 m	429 (75.1)	2083 (73.0)		1.12 (0.91, 1.38)	0.284	1.24 (1.00, 1.54)	0.048
Neuroblastoma							
>500 m (ref.)	67 (26.6)	772 (27.0)		1.00		1.00	
≤500 m	185 (73.4)	2083 (73.0)		1.03 (0.77, 1.38)	0.841	1.23 (0.91, 1.67)	0.175
Wilms tumor							
>500 m (ref.)	38 (26.7)	772 (27.0)		1.00		1.00	
≤500 m	105 (73.4)	2083 (73.0)		1.02 (0.70, 1.49)	0.928	1.14 (0.77, 1.68)	0.517
Retinoblastoma							
Unilateral							
>500 m (ref.)	16 (18.0)	772 (27.0)		1.00		1.00	
≤500 m	73 (82.0)	2083 (73.0)		1.68 (0.97, 2.91)	0.063	1.68 (0.96, 2.93)	0.071
Bilateral							
>500 m (ref.)	10 (31.3)	772 (27.0)		1.00		1.00	
≤500 m	22 (68.7)	2083 (73.0)		0.79 (0.37, 1.68)	0.539	0.72 (0.33, 1.57)	0.416
Hepatoblastoma							
>500 m (ref.)	11 (20.0)	772 (27.0)		1.00		1.00	
≤500 m	44 (80.0)	2083 (73.0)		1.49 (0.76, 2.90)	0.243	1.60 (0.80, 3.20)	0.181

Abbreviations: CI, confidence interval; m, meter; ref., reference; ^1^ Adjusted for birth year, birth weight, gestational age, maternal race/ethnicity, maternal age at delivery, and area-level poverty.

## References

[1] Gatta G., Ferrari A., Stiller C.A., Pastore G., Bisogno G., Trama A., Capocaccia R., Group R.W. (2012). Embryonal cancers in Europe. Eur. J. Cancer.

[2] Tulla M., Berthold F., Graf N., Rutkowski S., von Schweinitz D., Spix C., Kaatsch P. (2015). Incidence, Trends, and Survival of Children With Embryonal Tumors. Pediatrics.

[3] Phillips S.M., Padgett L.S., Leisenring W.M., Stratton K.K., Bishop K., Krull K.R., Alfano C.M., Gibson T.M., de Moor J.S., Hartigan D.B. (2015). Survivors of childhood cancer in the United States: Prevalence and burden of morbidity. Cancer Epidemiol. Prev. Biomark..

[4] Chu A., Heck J.E., Ribeiro K.B., Brennan P., Boffetta P., Buffler P., Hung R.J. (2010). Wilms’ tumour: A systematic review of risk factors and meta-analysis. Paediatr. Perinat. Epidemiol..

[5] Lohmann D. (2010). Retinoblastoma. Adv. Exp. Med. Biol..

[6] McLaughlin C.C., Baptiste M.S., Schymura M.J., Zdeb M.S., Nasca P.C. (2009). Perinatal risk factors for neuroblastoma. Cancer Causes Control.

[7] Spector L.G., Johnson K.J., Soler J.T., Puumala S.E. (2008). Perinatal risk factors for hepatoblastoma. Br. J. Cancer.

[8] Anderson L.M., Diwan B.A., Fear N.T., Roman E. (2000). Critical windows of exposure for children’s health: Cancer in human epidemiological studies and neoplasms in experimental animal models. Environ. Health Perspect..

[9] Health Effects Institute (2010). Traffic-Related Air Pollution: A Critical Review of the Literature on Emissions, Exposure and Health Effects.

[10] International Agency for Research on Cancer (1989). Diesel and gasoline engine exhausts and some nitroarenes. IARC Monographs on the Evaluation of Carcinogenic Risks to Humans.

[11] Population Reference Bureau 2017 World Population Data Sheet. http://www.prb.org/pdf17/2017_World_Population.pdf.

[12] Danysh H.E., Zhang K., Mitchell L.E., Scheurer M.E., Lupo P.J. (2016). Maternal residential proximity to major roadways at delivery and childhood central nervous system tumors. Environ. Res..

[13] Spycher B.D., Feller M., Roosli M., Ammann R.A., Diezi M., Egger M., Kuehni C.E. (2015). Childhood cancer and residential exposure to highways: A nationwide cohort study. Eur. J. Epidemiol..

[14] Steliarova-Foucher E., Stiller C., Lacour B., Kaatsch P. (2005). International Classification of Childhood Cancer, third edition. Cancer.

[15] Texas Department of State Health Services Texas Cancer Registry: Data Linkages. https://www.dshs.texas.gov/tcr/data/linkages.aspx.

[16] Texas Natural Resources Information System Maps & Data: Transportation StratMap, Version 2 (2006). http://tnris.org/data-download/#/statewide.

[17] U.S. Census Bureau (2007). TIGER/Line Files: Technical Documentation.

[18] Bunin G.R., Meadows A.T., Emanuel B.S., Buckley J.D., Woods W.G., Hammond G.D. (1989). Pre- and postconception factors associated with sporadic heritable and nonheritable retinoblastoma. Cancer Res..

[19] Dadvand P., Ostro B., Figueras F., Foraster M., Basagana X., Valentin A., Martinez D., Beelen R., Cirach M., Hoek G. (2014). Residential proximity to major roads and term low birth weight: The roles of air pollution, heat, noise, and road-adjacent trees. Epidemiology.

[20] Johnson K.J., Puumala S.E., Soler J.T., Spector L.G. (2008). Perinatal characteristics and risk of neuroblastoma. Int. J. Cancer.

[21] Yorifuji T., Naruse H., Kashima S., Takao S., Murakoshi T., Doi H., Kawachi I. (2013). Residential proximity to major roads and adverse birth outcomes: A hospital-based study. Environ. Health.

[22] Hertz-Picciotto I., Jusko T.A., Willman E.J., Baker R.J., Keller J.A., Teplin S.W., Charles M.J. (2008). A cohort study of in utero polychlorinated biphenyl (PCB) exposures in relation to secondary sex ratio. Environ. Health.

[23] Matz C.J., Stieb D.M., Davis K., Egyed M., Rose A., Chou B., Brion O. (2014). Effects of age, season, gender and urban-rural status on time-activity: Canadian Human Activity Pattern Survey 2 (CHAPS 2). Int. J. Environ. Res. Public Health.

[24] Friedrich P., Itriago E., Rodriguez-Galindo C., Ribeiro K. (2017). Racial and Ethnic Disparities in the Incidence of Pediatric Extracranial Embryonal Tumors. J. Natl. Cancer Inst..

[25] Heck J.E., Wu J., Lombardi C., Qiu J., Meyers T.J., Wilhelm M., Cockburn M., Ritz B. (2013). Childhood cancer and traffic-related air pollution exposure in pregnancy and early life. Environ. Health Perspect..

[26] Azary S., Ganguly A., Bunin G.R., Lombardi C., Park A.S., Ritz B., Heck J.E. (2016). Sporadic Retinoblastoma and Parental Smoking and Alcohol Consumption before and after Conception: A Report from the Children’s Oncology Group. PLoS ONE.

[27] Bunin G.R. (2004). Nongenetic causes of childhood cancers: Evidence from international variation, time trends, and risk factor studies. Toxicol. Appl. Pharmacol..

[28] McLaughlin C.C., Nasca P.C., Pastides H. (2008). Childhood Cancer. Fundamentals of Cancer Epidemiology.

[29] Herbstman J.B., Tang D., Zhu D., Qu L., Sjodin A., Li Z., Camann D., Perera F.P. (2012). Prenatal exposure to polycyclic aromatic hydrocarbons, benzo[a]pyrene-DNA adducts, and genomic DNA methylation in cord blood. Environ. Health Perspect..

[30] Perera F., Tang D., Whyatt R., Lederman S.A., Jedrychowski W. (2005). DNA damage from polycyclic aromatic hydrocarbons measured by benzo [a] pyrene-DNA adducts in mothers and newborns from Northern Manhattan, the World Trade Center Area, Poland, and China. Cancer Epidemiol. Prev. Biomark..

[31] Shimada T., Fujii-Kuriyama Y. (2004). Metabolic activation of polycyclic aromatic hydrocarbons to carcinogens by cytochromes P450 1A1 and 1B1. Cancer Sci..

[32] Moller P., Jacobsen N.R., Folkmann J.K., Danielsen P.H., Mikkelsen L., Hemmingsen J.G., Vesterdal L.K., Forchhammer L., Wallin H., Loft S. (2010). Role of oxidative damage in toxicity of particulates. Free Radic. Res..

[33] Wu W.S. (2006). The signaling mechanism of ROS in tumor progression. Cancer Metastasis Rev..

[34] Gilbert N.L., Woodhouse S., Stieb D.M., Brook J.R. (2003). Ambient nitrogen dioxide and distance from a major highway. Sci. Total Environ..

[35] Danysh H.E., Mitchell L.E., Zhang K., Scheurer M.E., Lupo P.J. (2017). Differences in environmental exposure assignment due to residential mobility among children with a central nervous system tumor: Texas, 1995–2009. J. Expo. Sci. Environ. Epidemiol..

[36] Lupo P.J., Symanski E., Chan W., Mitchell L.E., Waller D.K., Canfield M.A., Langlois P.H. (2010). Differences in exposure assignment between conception and delivery: The impact of maternal mobility. Paediatr. Perinat. Epidemiol..

[37] Heck J.E., Park A.S., Qiu J., Cockburn M., Ritz B. (2013). An exploratory study of ambient air toxics exposure in pregnancy and the risk of neuroblastoma in offspring. Environ. Res..

[38] U.S. Environmental Protection Agency Toxics Release Inventory (TRI) Explorer (2012 Dataset) [Internet Database]. http://www.epa.gov/triexplorer.

